# Novel Therapeutic Approaches to Liver Fibrosis Based on Targeting Oxidative Stress

**DOI:** 10.3390/antiox12081567

**Published:** 2023-08-05

**Authors:** Ana Blas-García, Nadezda Apostolova

**Affiliations:** 1 Departamento de Fisiología, Universitat de València, Av. Blasco Ibáñez, 15, 46010 Valencia, Spain; 2 FISABIO (Fundación para el Fomento de la Investigación Sanitaria y Biomédica de la Comunidad Valenciana), Av. de Catalunya, 21, 46020 Valencia, Spain; 3 CIBERehd (Centro de Investigación Biomédica en Red de Enfermedades Hepáticas y Digestivas), Instituto de Salud Carlos III, Monforte de Lemos, 3-5, 28029 Madrid, Spain; 4 Departamento de Farmacología, Universitat de València, Av. Blasco Ibáñez, 15, 46010 Valencia, Spain

**Keywords:** chronic liver disease, non-alcoholic fatty liver disease, mitochondria, NADPH oxidase, antifibrotic compounds, Nrf2, PPAR-γ, ferroptosis

## Abstract

Chronic liver disease (CLD) constitutes a growing global health issue, with no effective treatments currently available. Oxidative stress closely interacts with other cellular and molecular processes to trigger stress pathways in different hepatic cells and fuel the development of liver fibrosis. Therefore, inhibition of reactive oxygen species (ROS)-mediated effects and modulation of major antioxidant responses to counteract oxidative stress-induced damage have emerged as interesting targets to prevent or ameliorate liver injury. Although many preclinical studies have shown that dietary supplements with antioxidant properties can significantly prevent CLD progression in animal models, this strategy has not proved effective to significantly reduce fibrosis when translated into clinical trials. Novel and more specific therapeutic approaches are thus required to alleviate oxidative stress and reduce liver fibrosis. We have reviewed the relevant literature concerning the crucial role of alterations in redox homeostasis in different hepatic cell types during the progression of CLD and discussed current pharmacological approaches to ameliorate fibrosis by reducing oxidative stress focusing on selective modulation of enzymatic oxidant sources, antioxidant systems and ROS-mediated pathogenic processes.

## 1. Introduction

Chronic liver disease (CLD) is a major global health burden associated with important complications and high mortality [1], and it is expected to increase over the coming years given the high prevalence of non-alcoholic fatty liver disease (NAFLD), the most frequent form of CLD, which affects approximately 25% (or more) of the global population [2]. This metabolic disease, strongly associated with obesity and type 2 diabetes, encompasses a spectrum of pathologies ranging from simple steatosis to non-alcoholic steatohepatitis (NASH) with inflammation and/or liver fibrosis, and can progress to cirrhosis or hepatocellular carcinoma (HCC) [3].

Liver fibrosis, a well-orchestrated wound-healing response to chronic injury, is a dynamic process characterized by progressive accumulation of extracellular matrix (ECM) components, altered ECM degradation and distortion of liver parenchyma. However, when sustained over time, it impairs liver function and regeneration, and can lead to more advanced, life-threatening conditions [4]. Progressive fibrosis typically follows chronic liver damage caused by metabolic, infectious, cholestatic or drug-induced insults, and can develop into cirrhosis, liver failure or HCC [5]. The diagnosis and determination of the severity of liver fibrosis has crucial implications in the therapeutic management of CLD. The stage of liver fibrosis has traditionally been determined by liver biopsy; however, due to this method being invasive and susceptible to sampling errors, non-invasive (blood or bioimaging) tests have been gaining increasing interest in clinical care. Importantly, the severity of hepatic fibrosis is one of the strongest predictors to determine disease prognosis and quality of life in patients with CLD, especially those with NASH [6].

**Figure 1:**
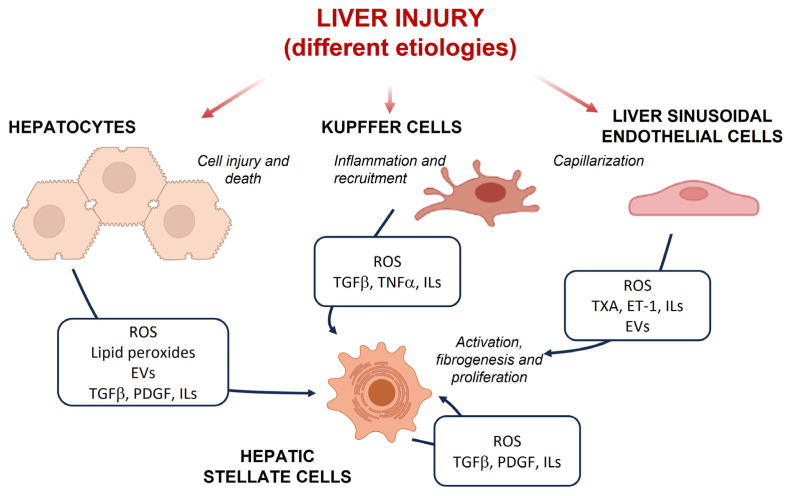
Intercellular crosstalk during liver injury. Liver insults of different etiology promote hepatocyte damage, capillarization of sinusoidal endothelial cells and activation of Kupffer cells and hepatic stellate cells, releasing warning signals such as pro-inflammatory cytokines, growth factors and reactive oxygen species (ROS), which contribute to the progression and perpetuation of liver damage. Abbreviations: ET-1, endothelin-1; EVs, extracellular vesicles; ILs, interleukins; PDGF, platelet-derived growth factor; TGFβ, transforming growth factor β; TNFα, tumor necrosis factor α; TXA, thromboxane.

Many different cell types are involved in the progression of liver fibrosis, including parenchymal and non-parenchymal hepatic cells, and immune cells recruited from peripheral blood. Once damaged, hepatocytes release warning signals, such as pro-inflammatory cytokines, growth factors and reactive oxygen species (ROS), which contribute to the progression and perpetuation of liver damage by activating pro-inflammatory and profibrogenic routes, and triggering a dynamic, highly integrated molecular and cellular process in collaboration with hepatic stellate cells (HSC), liver sinusoidal endothelial cells (LSEC) and different types of immune cells [7]. Activated HSC are the key effectors of fibrogenesis through increased deposition of collagen and ECM components, but they also release various mediators that contribute to the establishment of a profibrogenic environment, together with immune and inflammatory cells [8]. LSEC form the wall of hepatic sinusoids and play essential roles in hepatic homeostasis, which, besides regulation of the vascular tone, involves the maintenance of HSC quiescence and favors cellular crosstalk. They possess unique features, such as the presence of fenestra or pores, which are typically lost as liver injury progresses and ECM accumulates, in a process termed capillarization. Alterations of LSEC phenotype negatively affect other cells and contribute to fibrosis progression [9] (Figure 1). Increasing evidence highlights the role of immune cells and specific subsets of macrophages in the regulation of the profibrotic microenvironment of the liver. Upon liver injury, hepatic resident macrophages or Kupffer cells (KC) sense danger signals, interact with other hepatic cell populations and release chemokines that recruit circulating monocytes and other leukocytes. Once in the liver, monocytes differentiate into monocyte-derived macrophages which, together with KC, modulate the progression and resolution of hepatic injury and inflammation. This dual role is related to the high plasticity and diversity of macrophage subsets with a wide spectrum of different phenotypes [10].

Regression of liver fibrosis is associated with the elimination of activated HSC and the resorption of the fibrous scar. Activated HSC can be eliminated by one of three different mechanisms—apoptosis, senescence or reversion to an inactivated phenotype—but the relative contribution of these three pathways to fibrosis regression is still not clear. Usually, senescent stellate cells are cleared by natural killer cell-mediated cell death, and the inactivated cells have a phenotype that is similar to, but distinct from, quiescent HSC, by which they remain primed to respond to further liver injury [11]. In addition to direct inactivation of HSC, there are other mechanisms that can also improve the clinical situation and favor liver regeneration, such as inactivation and removal of specific macrophage and immune cell populations, ECM degradation, modulation of LSEC phenotype, and promotion of hepatocyte proliferation [12,13,14].

In this review, we summarize the importance of redox homeostasis in the liver and discuss novel therapeutic approaches to fibrosis based on targeting oxidative stress.

## 2. Mitochondria, Oxidative Stress and Liver Redox Homeostasis

**Figure 2:**
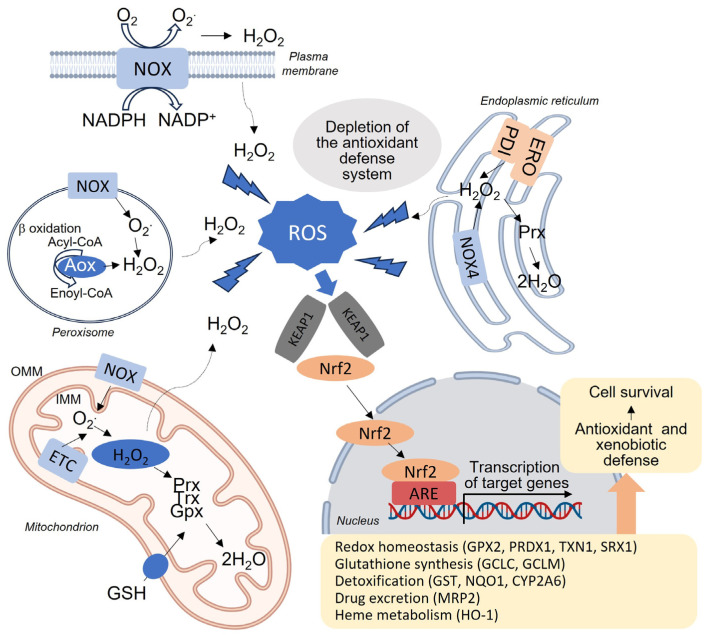
Major sources of ROS in hepatic cells. NADPH oxidase (NOX) enzyme, present in various cellular compartments, initiates the production of reactive oxygen species (ROS), primarily superoxide, although NOX4 has also been observed to generate hydrogen peroxide (H_2_O_2_). Within the process of cellular respiration, mitochondria transfer electrons to oxygen, resulting in ROS generation as a byproduct through the electron transport chain located in the inner mitochondrial membrane. Peroxisomes primarily generate ROS through peroxisomal β-oxidation. Acyl-CoA, converted to enoyl-CoA by acyl-CoA oxidase containing FAD, directly provides electrons to oxygen, resulting in the production of H_2_O_2_. Endoplasmic reticulum oxidoreductase 1 (ERO1) is an oxidoreductase residing in the endoplasmic reticulum responsible for facilitating disulfide bond formation, crucial for protein folding in nascent polypeptide substrates. This process involves electron transfer through protein disulfide isomerase (PDI), with oxygen serving as the final electron acceptor. The altered oxidative state of the cell triggers the activation of nuclear factor erythroid 2-related factor 2 (Nrf2), a master transcriptional regulator of the cellular response against oxidative stress. Under normal conditions, Nrf2 is bound to Keap1, and the Cul3 E3 ubiquitin ligase ubiquitinates Nrf2 for degradation by the proteasome. However, during oxidative stress, ROS modify the sensor cysteines in Keap1, leading to Nrf2 stabilization, accumulation, and translocation to the nucleus. In the nucleus, Nrf2 forms a heterodimer with sMaf and binds to the regulatory sequence known as the antioxidant response element (ARE), thereby activating the transcription of genes involved in antioxidant defense and phase II enzymes.

ROS are highly reactive molecules that are produced endogenously by many different enzymes and include a number of molecular species derived from oxygen, the main ones being the free radical superoxide anion (O_2_^•−^) and hydroxyl radical (•OH), and the non-radical hydrogen peroxide (H_2_O_2_). Although ROS can arise from all intracellular compartments, 90% are produced by mitochondria and, specifically, by their electron transport chain (ETC) [15]. Superoxide anions are the most prevalent ROS found in mitochondria [16]. In fact, a significant portion of the molecular oxygen consumed by these organelles, ranging from 0.2% to 2.0%, is converted to superoxide, which subsequently transforms into other types of ROS [15]. This is particularly noteworthy in the liver, where mitochondria are abundant due to the organ’s crucial metabolic role in the body. Each hepatocyte contains approximately 1000–2000 mitochondria. Additionally, there are numerous non-mitochondrial sources of ROS, such as NADPH oxidases (NOX), lipoxygenases, cyclooxygenases, peroxidases, xanthine oxidase, enzymes involved in peroxisomal fatty acid β-oxidation, and those involved in P-450 microsomal detoxification [17,18,19,20] (Figure 2).

NOX are particularly relevant in the pathophysiology of the liver [21,22]. In humans, the NOX family is composed of seven membrane-bound enzymatic complexes—namely, NOX1-5, DUOX1 and DUOX2—which vary in their tissue expression levels and activation mechanisms. NOX are cytoplasmatic generators of ROS, which is their only enzymatic activity, as opposed to other cellular ROS sources that generate ROS as secondary byproducts in addition to their main enzymatic function. NOX directly catalyze O_2_^•−^ production from O_2_ by transferring electrons from NADPH across biological membranes, thereby regulating many redox-sensitive signaling pathways. NOX1 and 2 mainly produce superoxide, whereas NOX4 directly produces H_2_O_2_. Loss of control of this coordinated ROS production is linked to the onset and progression of various pathologies, including liver diseases [23,24]. Expression of NOX isoforms differs among hepatic cell types; whereas hepatocytes, HSC and endothelial cells express NOX1, NOX2 and NOX4, KC mainly express NOX2 and the expression of NOX3 in the liver is still unclear. In addition, hepatocytes also express DUOX1 and DUOX2, but their significance remains elusive [22]. Enzymes that generate ROS may collaborate with other pathways associated with fibrosis to promote oxidative stress and the development of fibrogenesis. This can be observed through the interaction between NOX and Toll-like receptors (TLRs), which are transmembrane receptors responsible for recognizing microbial components in the innate immune response. The cytoplasmic tail of TLRs interacts with the carboxyl-terminal region of NOX, leading to the production of superoxide. Intriguingly, both NOX and TLRs are present in the liver, and the generation of ROS resulting from this interaction may have detrimental effects on liver cell function and contribute to the progression of fibrosis [25,26].

Generally, the amount of ROS under physiological conditions is moderate; however, under stressful conditions, ROS reach high concentrations that overwhelm the antioxidant capacity of the cells, causing what is known as “oxidative stress”. During this state, ROS are engaged in oxidative modification of cellular macromolecules, such as lipids, proteins and nucleic acids, which leads to a failure in the function of organelles including mitochondria, especially in hepatocytes [27]. Markers of oxidative stress include DNA damage marker 8-hydroxy-2′-deoxyguanosine (8-OHdG), lipid peroxidation products (thiobarbituric acid reactive substances or -TBARS-, malondialdehyde or -MDA-, 4-hydroxy-nonenal or -HNE-) and protein oxidation products (protein carbonyl, nitrotyrosine). Elevated oxidative stress markers have been observed in hepatic tissue and/or plasma of patients diagnosed with liver disease [28,29,30].

Given the toxicity of excess ROS, cells have developed diverse antioxidant systems involved in ROS detoxification, which comprise enzymatic and non-enzymatic components. Enzymatic components include superoxide dismutase (SOD), catalase (CAT), enzymes regulating glutathione (GSH) synthesis and redox cycle, such as glutathione peroxidase (GPx) and glutathione reductase (GR), and “redoxins” (peroxiredoxins, glutaredoxins, thioredoxins, and sulfiredoxins). The non-enzymatic antioxidant system includes small molecules, such as GSH, ubiquinone, lipoic acid, retinol (vitamin A), tocopherol (vitamin E) and ascorbic acid (vitamin C), which provide protection against radicals by accepting electrons in their structure [31]. In order to control the pro-oxidant effects of different external stimuli and internal (metabolic) pathways, the major stress-responsive transcription factors (such as activator protein-1 or -AP-1-, nuclear factor-κB or -NF-κB-, and nuclear factor E2-related factor 2 or -Nrf2-) are also redox sensitive [32]. Nrf2 is key in the oxidative stress-induced transcription of various antioxidant genes [33] and works in concert with small avian musculoaponeurotic fibrosarcoma oncogene homolog (MAF) proteins to recognize and bind antioxidant response elements (ARE) in promoters of antioxidant enzyme genes and phase 2 enzymes, such as glutathione S-transferase (GST), UDP-glucorosyl transferase, SOD, GPx, CAT, sulfiredoxin 1 and thioredoxin reductase 1 [34]. Nrf2 is inhibited by the Kelch-like ECH-associated protein 1 (Keap1), as this protein mediates Nrf2 proteasome-mediated degradation (Figure 2). Interestingly, elevated Nrf2 expression is associated with pathological ROS levels in liver biopsies of patients with NASH [35] and in diverse mouse NASH models; for instance, *Nrf2*-knockout mice display elevated susceptibility to NASH development [36,37]. Moreover, pharmacologic activation of the Nrf2-dependent antioxidant signaling pathway protects the liver in different oxidative stress models and diminishes liver steatosis and fibrosis in animals with diet-induced CLD [38,39].

Liver function is closely associated with the generation of ROS due to the metabolic and detoxification processes involved in alcohol and drug metabolism, which produce ROS as byproducts. Additionally, the liver plays a role in storing essential vitamins (A, B, D, E, and K), glycogen, and minerals like iron and copper, all of which participate in reactions that generate ROS. To counterbalance this, hepatocytes possess robust antioxidant defense systems. GSH, the primary redox buffer in the body, is particularly abundant in the liver, and replenishing its levels has demonstrated protective effects in liver disorders [34]. GSH is exclusively synthesized in the cytosol, necessitating specific carriers to transport GSH to different compartments, including mitochondria. In the liver, GSH is transported into mitochondria through the 2-oxoglutarate carrier (OGC), which exchanges GSH for 2-oxoglutarate (2-OG). Notably, the transport of GSH in hepatic disorders depends on membrane fluidity, which is influenced by fatty acid composition and cholesterol content. Increased membrane rigidity hampers this transport system, limiting the availability of mitochondrial GSH and its antioxidant protection.

Finally, ROS-mediated signaling plays a critical role in the liver, and disruptions in this process are linked to the development of liver diseases. This is partly attributed to the involvement of ROS in signal transduction, which regulates the production of inflammatory and immune factors.

## 3. Oxidative Stress in Liver Cell Populations during CLD

Increased and persistent oxidative stress is typical of CLD. Although in some circumstances it may not be enough to induce direct cell death, it can lead to a shift of redox homeostasis to a chronically deregulated state, which, in turn, upregulates different target genes involved in CLD progression, promoting inflammation, fibrogenesis and angiogenesis [40]. In this line, alterations in the pro-oxidant/antioxidant balance have been described in a variety of hepatic cell types during the progression of liver fibrosis.

### 3.1. Hepatocytes

The direct impact of the etiological agent on parenchymal cells usually results in oxidative stress associated with an enhanced intracellular generation of ROS and other redox-related intermediates. This effect not only contributes to the progression of fibrogenesis, but also to the perpetuation of chronic hepatocyte injury and cell death, and of the inflammatory response [41,42,43]. The alteration of the redox balance triggers different intracellular signaling pathways that induce changes in hepatocyte metabolism and/or lead to cell death. For instance, oxidative stress has been associated with endoplasmic reticulum (ER) stress and iron overload-mediated ferroptosis, as well as with the activation of the NLRP3 inflammasome and pyroptosis [44,45,46].

### 3.2. Hepatic Stellate Cells

Sustained production of ROS and redox-related reactive mediators by injured hepatocytes and activated non-parenchymal cells can directly modulate the phenotype and behavior of HSC to induce profibrogenic responses [47,48,49]. Furthermore, the generation of increased levels of ROS is a characteristic feature of activated HSC, associated with enhanced mitochondrial activity and the upregulation of NOX. Mitochondrial ROS increases have been clearly demonstrated during fibroblast activation in different tissues [50]; specifically, mitochondrial alterations have been observed in HSC proliferation and matrix production [51,52]. NOX activation in HSC has been extensively described as an important factor in ALD, NAFLD, cirrhosis and HCC [22,24,53]. In fact, classic activators of fibrogenesis, such as TGF-β, can activate the MAPK/ERK pathway and SMAD transcription factors, generating redox imbalances through the activation of several NOX enzymes, which subsequently enhances collagen production [54]. The crucial role of NOX-generated ROS in HSC activation has been repeatedly demonstrated by the inhibition or knockdown of these enzymes, which reduces the ability of profibrogenic stimuli to induce enhanced proliferation, contractility, fibrogenesis and the production of chemoattractant and inflammatory mediators [55,56].

### 3.3. Macrophages

The uptake of products of oxidative lipid peroxidation by these cells has been reported to result in suppressed pro-inflammatory cytokine production and reduced collagen deposition, as described in different models of liver fibrosis and isolated human macrophages [57,58]. Furthermore, pro-inflammatory hepatic macrophages have been shown to generate ROS through NOX2 [59], and NOX1-mediated cytokine production has been related to inflammation and tumorigenesis in murine models [60].

### 3.4. Liver Sinusoidal Endothelial Cells

The overabundance of lipids and insulin resistance that characterize NAFLD can lead to the downregulation of endothelial nitric oxide synthase (eNOS) activity and upregulation of inducible nitric oxide synthase (iNOS) and NOX1. These alterations induce nitro-oxidative stress via peroxynitrite production and, eventually, endothelial dysfunction, thus contributing to liver steatosis [61]. In addition to hepatocytes, LSEC also induce a lipotoxic response that contributes to ROS generation and progression to steatohepatitis. In fact, increased evidence of oxidative stress/ROS has been detected in LSEC both in preclinical models [62] and in patients with NAFLD and NASH [63], who displayed macrovesicular steatosis without any necro-inflammatory changes in the case of fatty liver, and the presence of macrovesicular steatosis with lobular inflammation with or without hepatocytes necrosis in the case of NASH.

## 4. Novel Therapeutic Approaches Targeting Oxidative Stress

Over the last few decades, continuous progress in basic, translational and clinical research has expanded our knowledge about the pathogenesis and potential regression of liver fibrosis. However, currently there are no approved drugs to halt or reverse liver fibrosis, and the only available treatment option is the eradication of the etiological agent (if possible) or liver transplantation for patients with advanced cirrhosis [64]. Generally, therapeutic approaches to liver fibrosis explored until now can be divided into agents that mediate hepatocyte protection, immune modulation or inhibition of HSC activation and elimination of the fibrotic scar. However, research in the field of antifibrotic drugs progresses slowly, and the antiviral and anti-inflammatory therapies used in clinical treatment do not effectively control or eradicate liver fibrosis, as they cannot prevent the deposition of ECM or promote its degradation.

The potential clinical severity of NASH has fueled the development of numerous studies aimed at identifying druggable targets. An important category of potential therapies is antifibrotic agents, especially those that directly interfere with fibrogenesis [65]. Although most of these agents have failed so far, there have been huge advances in the identification of the pathogenic mechanisms involved in fibrotic steatohepatitis, which has led to a highly diverse range of potential anti-NASH compounds being put forward [66], including those that modulate liver redox homeostasis. The beneficial effects of antioxidant supplementation preventing CLD progression in animal models have been demonstrated in many preclinical studies, as a consequence of in vivo attenuation of oxidative stress and/or lipid peroxidation [67,68,69]. Still, these preclinical approaches have not proved effective to significantly reduce fibrosis when translated into clinical trials [70,71,72].

Closely interacting with other cellular and molecular processes, increased oxidative stress triggers signaling pathways in different hepatic cell types and fuels the development of liver injury, fibrosis and carcinogenesis, especially in NASH [73]. Therefore, inhibition of ROS-mediated effects and modulation of major antioxidant pathways to counteract oxidative stress-induced injury have rapidly emerged as interesting targets to prevent or ameliorate liver damage. However, despite promising results in preclinical investigations, the therapeutic supplementation of generic antioxidant molecules in clinical trials of CLD has not generated undoubted results, probably due to the complexity of the interactions and compensatory mechanisms involved in redox homeostasis [74]. Hence, current pharmacological approaches to ameliorate fibrosis by reducing oxidative stress are focused on selective modulation of enzymatic oxidant sources or antioxidant systems with the purpose of undermining specific pathological processes in the disease’s progression. Some of these compounds are now in clinical development for use as antifibrotic agents, both in individual and in combined therapies [75].

### 4.1. Activation of Nrf2

The KEAP1/Nrf2 axis plays a major role in regulating cellular redox balance and is a prime therapeutic target for cancer and different chronic diseases, such as neurodegenerative diseases or NASH [76]; in fact, Nrf2 activity correlates with the grade of inflammation in liver samples from patients with NAFLD/NASH with different degrees of steatosis, fibrosis and inflammation (portal and lobular) including a pediatric cohort [77]. Several agents that activate Nrf2 are identified, either as approved drugs or under testing in clinical trials for different pathological conditions. However, it is not yet established whether the beneficial effects observed are specifically mediated by enhanced Nrf2 activity and the subsequent alleviation of oxidative stress [78,79].

Various studies in the liver have described the benefits of activating Nrf2-dependent responses in preclinical models of CLD and its potential for reducing fibrosis. For instance, functional studies in mice show that Nrf2 activation in hepatocytes resulting from specific KEAP1 deletion protects against fibrosis and cancer [77]. The potent activator of Nrf2 acetylenic tricyclic bis(cyano enone), TBE-31 has also been reported to diminish histologic evidence of liver fibrosis and expression of Col1a1 and α-Sma in high-fat-diet-with-fructose-fed mice with NASH in an Nrf2-dependent fashion [39]. S217879 disrupts the interaction between KEAP1 and Nrf2, thus triggering an enhancement of antioxidant pathways and the associated regulation of multiple genes involved in NASH disease progression. This effect ultimately led to the reduction of both NASH and liver fibrosis progression in mice [80]. Some of these effects may be related to the fact that the TGF-β/SMADs pathway, which is crucial in fibrosis, can be inhibited by Nrf2, resulting in reduced production of collagen and attenuated fibrosis. Such an effect has been reported in LX-2, an immortalized human cell line of HSC, and in a CCl_4_-induced fibrosis model treated with piperine, an alkaloid derived from black peppers [81]. Mechanistically, piperine was found to trigger Nrf2 nuclear translocation and the consequent transcription of antioxidant target genes, leading to a decrease in TGF-β1-induced production of ROS. 

### 4.2. NOX4/1 Inhibition

Due to the generation of ROS, NOX1, 2 and 4 promote hepatic fibrosis by participating in multiple processes, including HSC activation, proliferation, survival, and migration, hepatocyte apoptosis, enhancement of fibrogenic mediators, and mediation of an inflammatory cascade in both KC and HSC [82]. It is important to note that NOX-mediated ROS production has been shown to play an important role in fibrotic pathologies common to many chronic inflammatory diseases, as shown in cellular and animal preclinical models within the liver, kidney and lung [83,84,85,86].

Regarding cell type-related expression and functional differences, a variety of NOX isoforms are involved in the initiation of HSC activation and progression of fibrosis. Both NOX1 and NOX4 play crucial roles in the transdifferentiation of HSC [87]. While NOX4 contributes to fibrosis development directly, by inducing HSC activation, and indirectly, by triggering apoptotic cell death in hepatocytes [55,88], increased NOX1 expression in LSEC isolated from high-fat-diet-fed mice has been associated with enhanced ROS and peroxynitrite-induced hepatocellular injury and impaired hepatic microcirculation, thus accelerating cell death and NASH progression [62]. Notably, NOX2 has also been linked to LSEC premature senescence and associated fibrogenesis, as demonstrated by the attenuation of oxidative stress and fibrogenesis following the knockdown of NOX2 in a CCl_4_-induced liver fibrosis model [89].

The dual inhibitor of NOX1/4 setanaxib (GKT137831) is the first NOX inhibitor to progress to the stage of clinical trial. Its antifibrotic effects in different organs, due to dual NOX1/4 inhibition and/or redox modulation, are well supported by multiple preclinical studies [86]. Specifically, setanaxib has been described to replicate the beneficial effects seen in multiple NOX1/4 KO models of liver fibrosis, reducing oxidative stress, hepatocyte apoptosis, inflammation and fibrosis in different types of CLD, including NASH, bile duct ligation and CCl_4_ treatment [88,90]. In line with these results, treatment with this dual inhibitor diminished ROS production and expression of profibrogenic and proliferative genes in primary human and mouse HSC, even when they were stimulated with PDGF or LPS [87]. Although this evidence is promising, the precise role of NOX1/4 in fibrotic pathologies and the selective inhibitory action of setanaxib have not been fully elucidated. The use of selective inhibitors to target specific NOX isoforms (such as NOX1 and/or NOX4) is a promising approach to treating liver fibrosis, due to the prevention of HSC activation and protection against hepatocyte injury, although further work is needed to fully confirm the clinical safety of these compounds. Moreover, understanding the molecular pathways involved in NOX-mediated HSC activation and fibrogenesis could provide new insight for developing novel antifibrotic treatments.

### 4.3. Peroxisome Proliferator-Activated Receptor γ (PPAR-γ) Activation

PPAR-γ and other PPARs are key regulators of metabolic diseases, playing crucial roles in lipid metabolism, regulation of energy balance, inflammation and fibrosis [91]. PPAR-γ is mainly expressed in adipose tissue, but also in other cells and tissues such as immune cells and the liver itself, and it is known to participate in the pathogenesis of NAFLD, in which it plays multifaceted roles. On the one hand, this transcription factor promotes liver adipogenesis and steatosis, increasing fatty acid uptake and triglyceride synthesis in hepatocytes, and the upregulation of adipogenic genes via sterol regulatory element-binding protein 1c (SREBP-1c) [92]. On the other hand, PPAR-γ has been proven to exert hepatoprotective effects by preventing pro-inflammatory polarization of macrophages, thereby reducing oxidative stress and reversing the activation of HSC [93,94,95,96]. To mitigate oxidative stress in the liver, PPAR-γ not only directly binds to the PPAR-γ response element on the Nrf2 promoter to promote its expression, but also regulates ROS production by inhibiting iNOS or enhancing the activity of eNOS [97].

PPAR-γ is a promising therapeutic target for fibrosis and, especially, for NAFLD. The development of drugs that can balance the beneficial and adverse effects of PPAR-γ is sure to bring new hope to patients with this disease.

### 4.4. ER Stress and UPR

The ER is the intracellular organelle responsible for synthesis, folding, trafficking, and maturation of proteins, in addition to many other important functions such as triglyceride and cholesterol synthesis, storage of Ca^2+^ and xenobiotic metabolism. The ER is also a source of ROS and actually, after mitochondria, it is the organelle that consumes the most molecular oxygen because it contains several oxygenases. ROS generation in the ER occurs through the action of several enzymes, including the protein disulfide isomerase (PDI)/endoplasmic reticulum oxidoreductase 1 (ERO1) enzymatic hub, NOX or cytochrome p450, in tissues such as the liver (Figure 2). While most NOX members are located in the plasma membrane and produce superoxide as primary ROS, NOX4, an ER-resident enzyme, faces the ER lumen and releases hydrogen peroxide. Also, the redox environment in the ER is oxidative compared with that of the cytosol, and ER function is very susceptible to changes in the redox status [98]. Under pathophysiological conditions, the folding capacity of the ER is undermined, thereby impacting the rate of protein synthesis and folding, leading to what is known as ER stress. This triggers a homeostatic adaptive response which has been termed the unfolded protein response (UPR). The insufficiency of the UPR to meet the increased folding needs of the cell activates a pathologic response resulting in lipogenesis, inflammation, and activation of apoptotic pathways. In this regard, ER stress very often occurs concomitantly with mitochondrial dysfunction and oxidative stress making it very difficult to untangle this complicated triad. It is widely known that UPR/ER stress plays an important role in virtually all liver diseases, and this has been studied more abundantly in hepatocytes which are rich in ER [99,100]. Thus, modulators of ER stress/UPR are of interest for developing treatments of liver diseases. Ursodeoxycholic acid (UDCA) and tauroursodeoxycholic (TUDCA), and sodium phenylbutyrate (4-PBA) are low-molecular-weight chemical chaperones that promote protein folding and assembly and are FDA-approved agents employed to treat primary biliary disease and urea-cycle disorder, respectively. In addition to these drugs, multiple molecules have been shown to be hepatoprotective in animal models through their action on the ER. For example, berberine, a natural plant alkaloid, has been shown to prevent the progression from steatosis and steatohepatitis by reducing ER stress [101]. Thus, several pharmacological interventions may be used to target sustained UPR for the treatment of liver diseases, but their specific impact on ROS production requires further investigation.

### 4.5. Mitochondrial Dysfunction and Mitochondrial Quality Control

Mitochondrial function and its specific aspects (ROS generation, mitochondrial dynamics and mitochondrial quality control) is crucial for the understanding of liver disease pathogenesis and its treatment. A very recent work has highlighted the relevance of mitochondrial oxidative stress, mtDNA damage, and related alterations in mitochondrial function and dynamics in advanced fibrosis in chronic hepatitis B and NASH [43]. Such research suggests that targeting mitochondrial function is undoubtedly an important approach for the treatment of liver disease and, specifically, liver fibrosis. There is plenty of evidence of the beneficial effect of antioxidants (including mitochondria-targeted ones) for treatment of liver disease in experimental animals. In this context, coenzyme Q10 (CoQ10), also known as ubiquinone, is an important component of the mitochondrial ETC and is widely used as an antioxidant supplement. Oral administration of CoQ10 was shown to reduce oxidative stress and liver fibrosis in a rat model of poor maternal nutrition [102] and in a mouse model of dimethylnitrosamine-induced liver fibrosis [103]. The mitochondria-targeted version of CoQ10, mitoquinone (MitoQ), attenuated CCl_4_-induced liver fibrosis in mice [104,105], and this effect involved the regulation of the JNK/YAP pathway [106]. Similarly, in cirrhotic rats, MitoQ decreased hepatic oxidative stress, thus improving liver inflammation and fibrosis, and preventing apoptosis [69], and was found to reduce intrahepatic vascular resistance [52]. Both CoQ10 and MitoQ have been assayed in clinical trials with patients suffering from CLD; however, their usefulness in clinical practice remains questionable and needs to be assessed further [107].

Damaged mitochondria are selectively removed from the cell through an evolutionarily conserved, selective degradation process involving macroautophagy and known as mitophagy. Importantly, deficient mitophagy in hepatocytes has been associated with the development of liver disease, so targeting mitophagy presents itself as a promising therapeutic target. A protective effect of mitophagy—regulated by the mTOR signaling pathway—has been described in a rat model of liver fibrosis associated with selenium deficiency [108]. Similarly, melatonin was seen to enhance mitophagy and mitochondrial biogenesis while diminishing liver fibrosis in rats [109]. Mitophagy was also found to be enhanced as a part of the hepatoprotective effect of MitoQ in fibrotic experimental animals [69,105].

Another important aspect is mitochondrial dynamics (mitochondrial fission and fusion), which can influence, and be influenced by, mitochondrial function and ROS generation. Also, mitochondrial fission usually precedes mitophagy. This process can be regulated by oxidative stress and is considered a negative regulator of mitochondrial ROS signaling through selective degradation of dysfunctional mitochondria. Possible novel targets for CLD have been put forward based on mitochondrial ROS/dynamics/mitophagy interplay. For example, the DNA-dependent protein kinase catalytic subunit (DNA-PKcs) promotes alcohol-related liver disease in mice by activating dynamin-related protein 1 (Drp1)-related mitochondrial fission and repressing FUNDC1-required mitophagy [110].

While stimulation of mitophagy in hepatocytes is clearly hepatoprotective, the role in liver fibrosis of mitophagy in other cell types in the liver seems to be much more complex, and therefore its pharmacological manipulation should be considered with caution. For example, induction of liver fibrosis by CCl_4_ is associated with increased expression in Kupffer cells of T-cell immunoglobulin domain and mucin domain-4 (TIM-4), a protein selectively expressed on antigen-presenting cells, together with augmented ROS production, mitophagy and TGF-β1 secretion. TIM-4 interference was shown to lead to liver fibrosis resolution, while inhibiting Akt1-mediated ROS production and consequently suppressing PINK1, Parkin and LC3-II/I activation [111].

Finally, mitochondria-derived damage-associated molecular patterns (mito-DAMP), in which mtDNA is the major active component, are a promising target to be exploited for the treatment of liver disease with fibrosis. These molecules enhance the interconnection among different hepatic cell types as part of the pathogenic process. In this regard, it has recently been demonstrated that mito-DAMP released from injured mitochondria in hepatocytes directly activate HSC in a liver fibrosis resistant/susceptible mouse strain system. In addition, circulating mito-DAMP were markedly increased in two cohorts of patients with NASH and liver fibrosis; the majority of them had active NASH, defined histopathologically as NAFLD activity score (NAS) ≥ 4, and various degrees of fibrosis ranging from F0 (no fibrosis) to F4 (cirrhosis) [108]. Of note, circulating mtDNA levels were increased in patients with active NASH (NAS score 4–8, versus NAS score 0–3) and particularly in those with significant histological signs of fibrosis (F2–4) on biopsy, compared to patients with minimal/no fibrosis [112]. Interestingly, mito-DAMP have been studied in cell types other than hepatocytes. For instance, in macrophages, liver fibrosis triggers the cytosolic release of self-mtDNA. X-box binding protein 1 (Xbp1) has been reported to control cGAS/STING/NLRP3 activation by the regulation of macrophage mtDNA cytosolic leakage, via the downregulation of BNIP3-mediated mitophagy. This is related to the fact that myeloid-specific Xbp1 deficiency or pharmacological inhibition of Xbp1 ameliorates liver fibrosis in mice [113].

In summary, intervention in mitochondrial pathophysiology represents a promising approach to the therapeutics of liver fibrosis; however, as occurs with most of the treatments tested in this area, there is a clear need for targeted, organ- and cell-type specific methods.

### 4.6. Regulation of Cell Death: Ferroptosis and Pyroptosis

#### 4.6.1. Ferroptosis

Ferroptosis is an iron-dependent form of regulated cell death characterized by the alteration of the cellular redox state and accumulation of large amounts of lipid peroxides. Interestingly, it has been related to both the development and attenuation of liver fibrosis [114] due to its different actions in distinct cell types. On one hand, activation of ferroptosis has been associated with hepatocyte cell death and injury progression in different CLD, including NAFLD and ALD [115]. Different studies have showed that hepatocyte ferroptosis caused by iron overload and lipid peroxidation may be involved in the pathological process of NAFLD, aggravating the inflammatory response, oxidative stress and cell damage in the early stages of NASH [116,117,118]. Equally, the role of ferroptosis in ALD has been demonstrated in preclinical models of this disease, which showed that alcohol-induced accumulation of ROS and lipid peroxidation were counteracted by ferroptosis inhibitors [119]. On the other hand, ferroptosis can be considered a new strategy to eliminate activated HSC and ameliorate liver fibrosis [120,121]. In fact, several compounds are thought to exert antifibrotic effects by inducing this type of cell death in HSC in in vivo and in vitro preclinical models. In this line, induction of ferroptosis in HSC by sorafenib was found to remarkably alleviate liver fibrosis characterized by decreased collagen deposition [122]. Similarly, berberin has been shown to inactivate HSC and inhibit ECM production via ferroptosis in vitro and in vivo. Regarding the mechanisms involved, ferritin was modulated by berberin-mediated autophagy/ROS and the ubiquitin–proteasome system (UPS) pathway, triggering redox-active iron overload and iron homeostasis disruption. As a consequence, additional ROS are generated, inevitably leading to the ferroptosis of HSC [123]. Consequently, one of the main concerns of this therapeutic strategy is that ferroptosis-inducing agents may have deleterious effects on hepatocytes and macrophages; however, sorafenib is incapable of triggering redox-active iron overload, lipid ROS accumulation, or MDA production in macrophages and primary hepatocytes [122].

#### 4.6.2. Pyroptosis

Pyroptosis is a type of inflammatory cell death that results from activation of the NLRP3 inflammasome and involves cell lysis by the pore-forming protein gasdermin D (GSDMD). This inflammasome is a multiprotein complex expressed in hepatocytes, HSC, macrophages, and LSEC, and can be activated by a wide variety of stimuli, such as gut-derived PAMP, cell damage-induced DAMP, TNF-α, cholesterol crystals, and ROS. Once activated, it releases the pro-inflammatory cytokines IL-1β and IL-18, inducing cell death by pyroptosis and exacerbating inflammation, monocyte and neutrophil recruitment, and fibrosis [124,125]. Oxidative stress plays an important role in pyroptosis as ROS production is necessary, not only for the priming and induction of the NLRP3 inflammasome, but also for GSDMD pore formation in the plasma membrane. Consequently, attenuation of ROS production by antioxidant treatments is a promising approach for preventing inflammasome-mediated inflammation and fibrosis in the liver. In this line, antioxidants have been reported to decrease ROS and NLRP3 inflammasome activation in several models of acute and chronic liver injury [126]. Interestingly, some of these beneficial effects are related to an Nrf2-mediated response and, thus, Nrf2 inducers have been suggested as liver protective compounds via the prevention of pyroptosis [127].

## 5. Conclusions and Future Perspectives

Effective and specific antifibrotic drugs are urgently needed to reduce the burden of CLD worldwide. Given the importance of oxidative stress in the pathogenesis of CLD, the use of antioxidative agents for the treatment of liver fibrosis has attracted much attention. However, the lack of significant fibrosis reduction in clinical trials using general antioxidants highlights the need for selective and cell/tissue-specific targeting of oxidant-modulating drugs. The vast majority of studies to date have centered on hepatocytes; however, the roles of other cell types in the liver and the possibility of their therapeutic manipulation are increasingly becoming the focus of research. Although few LSEC interventions have been developed so far, improving their health appears to prevent CLD progression and complications. Among the different cellular compartments, mitochondria stand out as a promising target in antifibrotic drug development. Importantly, the variations in liver fibrosis evaluation methods across various studies and, particularly, regarding liver fibrosis regression pose a challenge to the interpretation and comparison of the published findings.

Considering the complexity of liver fibrosis and the involvement of different cell types with high plasticity, combination therapies may be the key to achieving more effective therapy by enhancing the effects of antifibrotic compounds with diverse targets within the disease’s pathogenesis, such as oxidative stress, HSC inactivation and ECM degradation.

## Data Availability

Data are available in the original articles cited in the present review.
